# Isolation of Bacteria with Potential Plant-Promoting Traits and Optimization of Their Growth Conditions

**DOI:** 10.1007/s00284-020-02303-w

**Published:** 2020-12-23

**Authors:** Mohammad Yaghoubi Khanghahi, Sabrina Strafella, Ignazio Allegretta, Carmine Crecchio

**Affiliations:** Department of Soil, Plant and Food Sciences, University of Bari Aldo Moro, Via Amendola 165/A, 70126 Bari, Italy

## Abstract

This research aimed at investigating the isolation and identification of bacterial strains with biological nitrogen*-*fixing capability and phosphate, potassium, and zinc solubilization activities from a durum wheat field under two different tillage practices including 10 years of conventional tillage (CT) and no-tillage (NT) practices. Attempts were also extended to estimate their relative abundances in the soil as well as to develop accurate mathematical models in determining the effect of different temperatures, NaCl concentrations and pH on the growth, and activity of selected isolates. Twelve effective bacterial strains, including *Pseudomonas*, *Acinetobacter*, and *Comamonas* genera, were identified with a great potential to solubilize the insoluble forms of phosphate (from 11.1 to 115.5 mg l^−1^ at pH 8), potassium (from 32.2 to 35.6 mg l^−1^ at pH 7), and zinc (from 1.11 to 389.90 mg l^−1^ at pH 9) as well as to fix N_2_ gas (from 19.9 to 25.2 mg l^−1^). To our knowledge, this is the first report of the ability of *Comamonas testosteroni* and *Acinetobacter pittii* to fix nitrogen and to *solubilize insoluble potassium compound, respectively.* Three families, *Moraxellaceae*, *Pseudomonadaceae,* and *Comamonadaceae,* showed a higher percentage of abundance in the NT samples as compared to the CT, but only significant difference was observed in the relative abundance of *Pseudomonadaceae* (*P* < 0.01)*.* These strains could be definitively recommended as inoculants to promote plant growth in the wide ranges of pH, salinity levels (with maximum growth and complete inhibition of growth from 0.67–0.92% to 3.5–9.3% NaCl, respectively), and temperatures (2.1–45.1 °C).

## Introduction

Wheat cultivation is the main farmers’ income source in the Mediterranean area. This plant requires about 15–30 kg nitrogen (N), 3–5 kg phosphorus (P), 3–6 kg potassium (K), and 0.03–0.06 kg Zinc (Zn) per ton of grain yield [1, 2]. Since total grain production of durum wheat (*Triticum durum* Desf.) in Italy, one of the leaders in the world is around 42.46 million tons in 2018 [3], it can be easily estimated how many megatons of these nutrients are needed for wheat cultivation as an annual removal from soil. On the other hand, much more chemical fertilizers are generally applied to supply essential nutrients to the soil–plant system throughout the world, because some elements once applied are not easily available to the plant [4]. For example, less than half (10–40%) of the applied nitrogen in the field is effectively absorbed by plants, and 60–90% of chemical N fertilizers are generally lost by nitrate leaching or ammonia volatilization [5]. Also, P absorption by the wheat plant is only about 20% of the chemical fertilizer applied at the first year [2]. In addition, some elements dissolve relatively slowly in soil, taking too much time to supply adequate amounts required for plant growth [4]. Therefore, farmers usually apply huge amounts of chemical fertilizers, which can result in negative impact on human health and the environment such as soil pollution and/or greenhouse-gas generation [6]. This problem is associated with costs and availability of chemical fertilizers which are real issues of today’s agriculture [7].

Therefore, interest has grown in eco-friendly and cost-effective agro-technologies to enhance crop production and reduce the chemical fertilizers input while minimizing negative effects on the environment and food [7, 8]. Soil microbial composition and abundance, as a component of soil ecosystem, play an important role in nutrient availability in soil and nutrient status in the plant [9]. The use of microbial inoculants or naturally occurring plant growth-promoting bacteria (PGPB) in sustainable agriculture is becoming a more widely accepted practice in many parts of the world [10, 11]. PGPB promote plant growth usually by direct and indirect mechanisms including biological nitrogen fixation, organic matter mineralization, phytohormones production, e.g., auxins, cytokinins, and gibberellins, biological control against soil borne pathogens, cellulose degradation, starch hydrolysis, production of hydrogen cyanide, antibiotic, siderophore, and certain volatile organic compounds [10]. However, the PGPB efficiency is strongly affected by environmental factors such as salinity, pH, and temperature due to changing their survival, growth, and activity to stressful conditions [6, 11]. The majority of credible group of PGPB belongs to genera *Acinetobacter*, *Azadirachta*, *Azotobacter*, *Azospirillium*, *Bacillus*, *Pantoea*, *Pseudomonas*, *Rahnella*, *Rhizobium*, *Serratia,* and *Streptomyces* sp. [6, 11–13]. In this regard, Bakhshandeh et al. [6] isolated several bacterial strains with phosphate solubilization activity such as *Pantoea ananatis*, *Rahnella aquatilis*, and *Enterobacter* sp. which were able to solubilize, respectively, 172, 263, and 254 µg ml^−1^ of Ca_3_(PO_4_)_2_ after 5 days of growth at 28 °C and pH 7.5. Potassium solubilization activity of *Pantoea agglomerans*, *Rahnella aquatilis,* and *Pseudomonas orientalis* were reported equal to 35, 76, and 56 µg ml^−1^, respectively, of K in Aleksandrov liquid medium after 21 days of incubation [11]. The Zn solubilization by *Rhizobium* sp. and *Agrobacterium tumefaciens* in tris-mineral medium reached the highest value at pH 9 equal to 72 and 51 mg l^−1^, respectively [13].

Since the knowledge of the native beneficial bacterial population, the irrelative abundance in the soil, identification, characterization, and optimization of their growth conditions are important for understanding the performance and diversity of indigenous bacteria in the soil of specific crops, this research aimed to (i) isolate and identify bacterial isolates with biological nitrogen*-*fixing capability and phosphate, potassium, and zinc solubilization activities from durum wheat field; (ii) develop accurate mathematical models to determine the effect of temperature, NaCl concentration, and pH on the growth and activity of selected isolates; (iii) evaluate, by metagenomic approaches, if tillage/no-tillage management affects the presence of these potentially beneficial bacteria.

## Materials and Methods

### Sample Collection

The sampling was conducted in a Mediterranean soil ecosystem at Lavello (Southern Italy, Basilicata region, located at 41°03′ N, 15°42′ E, altitude of 180 m above the average of sea level, with an average of 570 mm and 14.5 °C of long-term annual rainfall and temperature, respectively). Since soil tillage managements have complex effects on soil physical, chemical, and biological properties which could subsequently affect PGPB activity in soil [14], the potentially beneficial bacterial isolation was performed in a field under two different tillage practices including 10 years of conventional tillage (CT) and no-tillage (NT) practices. Both plots have been cultivated for 10 years with a biennial rotation of durum wheat and legumes including broad bean (*Vicia faba* L.), green pea (*Pisum sativum* L.), and chickpea (*Cicer arietinum* L*.*). In both plots, straw and root residues of durum wheat and legumes have been left on the fields. Three composite soil samples from each plot were collected after harvesting of the durum wheat in 2018. A minimum of five sub-samples were taken randomly by hand auger and combined into one composite sample. Fine fraction passing a 2 mm sieve was collected and transported refrigerated at 4 °C.

### Isolation of Bacterial Strains

5 g of each sample was weighed and then homogenized in a 45 ml sterile Ringer solution and 5 ml pyrophosphate. Microbial communities were desorbed from soil by sonication. Serial dilution (10^−2^ to 10^−6^) of samples prepared and then poured on Nutrient Agar (NA) plates supplemented with 1% (w/v) cycloheximide; the plates were incubated at 30 °C for 48 h [15]. The streaking technique was used on NA plates to get single colonies for further investigation.

### Isolation of Nitrogen-Fixing Bacteria

Nitrogen fixation capability of isolates was measured in N-free medium [15] containing 5 g malic acid, 0.5 g KH_2_PO_4_, 0.2 g MgSO_4_·7H_2_O, 0.1 g NaCl, 4.5 g KOH, 1.4% agar, 0.02 g CaCl_2_, 2 ml micronutrient solution (l^−1^; 0.04 g CuSO_4_·5H_2_O, 1.2 g ZnSO_4_·7H_2_O, 1.4 g H_3_BO_3_, 1 g Na_2_MoO_4_·2H_2_O, 1.175 g MnSO_4_·H_2_O), 2 ml bromothymol blue (0.5% sol 0.2 mol l^−1^ KOH), 1 ml Fe-EDTA (1.64%), 4 ml vitamins solution (100 ml^−1^; 10 mg biotin, 20 mg piridixol-HCl) in 1000 ml deionized water, and pH was adjusted to 7.0. A loop full of bacterial overnight growth in Nutrient Broth (NB) was spread on solid N-free medium plates. The experiments were made in triplicate, and a blue halo zone after 7 days of incubation at 30 °C was considered as qualitative evidence of N_2_ gas fixation.

### Selection of Effective Phosphate, Potassium and Zinc-Solubilizing Strains

Phosphate-solubilizing abilities of all isolated colonies were measured on Pikovskaya agar (PVK) medium [16] containing 10 g glucose, 5 g Ca_3_(PO_4_)_2_, 0.5 g (NH_4_)_2_SO_4_, 0.2 g NaCl, 0.1 g MgSO_4__7H_2_O, 0.2 g KCl, 0.5 g yeast extract, 0.0003 g MnSO_4__H_2_O, 0.0003 g FeSO_4__7H_2_O, 10 g agar (1000 ml deionized water), and pH 7.0.

Potassium-solubilizing ability was also determined on modified Aleksandrov agar medium containing 3.5 g Glucose, 0.5 g MgSO_4_·7H_2_O, 0.1 g CaCO_3_, 0.0005 g FeCl_3_, 2.0 g Ca_3_PO_4_, 1.0 g insoluble mica powder as potassium source, and 15.0 g agar (1000 ml deionized water and pH was adjusted 7.0) [17].

Tris-mineral agar medium was used to evaluate zinc-solubilizing ability containing 10.0 g d-glucose, 1.0 g (NH_4_)_2_SO_4_, 0.2 g KCl, 0.1 g K_2_HPO_4_, 0.2 g MgSO_4_, 1.244 g zinc oxide (0.1% Zn concentration) in 1000 ml deionized water at pH7.0 [18].

P, K, and Zn solubilization and N fixation efficiency calculated (Eq. 1) by incubating the isolates on agarized PVK, Aleksandrov, Tris-mineral, and N-free mediums at 29 °C for 7 days [19].

1 $${\text{NE}} = \left( {\frac{{{\text{HZ}}}}{C}} \right) \times 100,$$

where NE is the nutrient solubilization or fixation efficiency, HZ the diameter of the solubilization halo zone, and *C* is the diameter of the colony.

### Quantitative Estimation of P, K, and Zn Solubilization and N Fixation

Based on the results of the plate assay, four isolates which showed the best solubilization of phosphate, potassium, and zinc, as well as fixation of nitrogen gas, were subjected to quantify the amount of P, K, Zn, and N in the liquid medium. Accordingly, inoculated and non-inoculated (control) liquid mediums were incubated at 29 °C in a shaker at 200 rpm for 14 days. Total-reflection X-ray fluorescence spectrometry (TXRF) technique was used to analyses solubilized P, K, and Zn in the supernatant of the bacterial cultures, as fully described by Yaghoubi et al. [13], using an S2Picofox TXRF Spectrometer (Bruker Nano GmbH, Berlin, Germany). Also, the total N of liquid samples was determined by the Kjeldahl (Model UDK 149 Automatic Kjeldahl Distillation Unit, VELP Scientifica, Italy) method.

### Effect of pH on Solubilizing Ability of PGPB Isolates

The PVK, Aleksandrov, and Tris-mineral mediums were used to estimate the effect of five pH values (6, 7, 8, 9, and 10) on P-, K-, and Zn-solubilizing ability of selected isolates, respectively, as previously described.

### Indole Acetic Acid (IAA) Production

In vitro production of IAA for each isolate was determined colorimetrically following the method of Gordon and Weber [20]. The bacteria were grown in NB media supplemented with l-tryptophan (1 mg ml^−1^) at 30 °C for 72 h, and then the supernatants were collected by centrifugation at 5500 rpm for 15 min. One ml of the culture filtrate was allowed to react with 4 ml of Salkowsky reagent (1 ml 0.5 M FeCl_3_; 30 ml H_2_SO_4_ 98%; 50 ml distilled water) at room temperature in the dark for 20 min. The optical density (OD) of solution was read at 535 nm in a spectrophotometer (Model Ultrospec 4000, Pharmacia Biotech Inc. USA), and the amount of IAA produced was calculated by comparing with the standard curve prepared with pure IAA.

### Bacterial DNA Extraction and 16S rRNA Gene Sequencing

Genomic DNA of isolates was extracted using the DNeasy Blood & Tissue Kit (Qiagen, Hilden, Germany), and the 16S rRNA gene PCR amplification was performed by the universal primers 357F and 1401R, corresponding to the position 341–357 and 1385–1401, respectively, of the 16S rRNA gene sequence of *Escherichia coli* [21]*.* All PCR amplifications were carried out in 25 μl reactions containing 100 ng of total DNA, 10 mM of each 2′-deoxynucleoside 5′-triphosfate (dNTP), 3 U of Taq DNA polymerase (EuroTaq; EuroClone), and 2.5 mM MgCl_2_ using a MyCycler™ thermal cycler (Bio-Rad Laboratories Inc.). The 1060 bp amplicons were purified using the Wizard® SV Gel and PCR Clean-Up System kit (Promega, USA) and sequenced from both ends by Eurofins Genomics (Milan, Italy). The 16S rRNA sequences were aligned using the BLASTn tool against the NCBI database (www.ncbi.nlm.nih.gov) to identify the bacterial strains.

### Soil DNA Extraction and Bacterial Community Analyses

DNA was extracted from soils using the FastDNA® SPIN Kit for Soil (MP Biomedicals, Solon, CA, USA). Aliquots of the extracted DNA were sent to the IGA Technology Service in Udine (Italy) for metagenomic analyses; the sequencing was performed through the MiSeq Illumina system platform. QIIME software (version 1.9.1) was used to perform bacterial community analysis as fully described by Kuczynski et al. [22]. Amplification of the variable V3 and V4 regions of the 16S rRNA gene was performed in two steps, including a PCR amplification using locus-specific primers and the flow-cell binding sequence (Nextera XT Index Kit, FC-131-1001/FC-131-1002). Aligned sequences were clustered into operational taxonomic units (OTUs) with a 97% similarity cut-off after removing the singletons or non-bacterial OTUs. Bacterial *α*-diversity measures (Good’s coverage estimator, Chao1 index, Shannon index, and Simpson index) were computed based on 10,000 reads per samples. A cut-off of 50% of the target sequencing coverage was considered for rarefaction curves endpoints and normalization of counts. A minimum confidence threshold of 50% was also applied to assign the bacterial taxonomy using the Ribosomal Database Project (RDP) Naïve Bayesian classifier.

### Modeling Salt Stress Tolerance of the Selected Bacteria

Different NaCl concentrations including 0.5 (control treatment), 1, 1.5, 2, and 3% (w/v) were used to estimate the bacterial growth under salt stress (NaCl) in Nutrient Broth (NB) medium. The bacterial growth was evaluated by measuring the turbidity at 600 nmin 0, 5, 9, 15, 20, 24, 29, 33, and 48 h of incubation at 29 °C. A logistic equation (Eq. 2) was used to predict the influence of NaCl concentration on isolates growth. Equation (3) is used to estimate the rate of absorbance increase over time (absorbance per hour) at different NaCl concentrations of NB medium [13, 23].

2 $${\text{Ab}} = \frac{{{\text{Ab}}_{{{\text{max}}}} }}{{1 + x/x50^{s} }},$$

where Ab is absorbance at concentration *x*, Ab_max_ is maximum absorbance, *x*_50_ is concentration of NaCl required for 50% inhibition of the maximum absorbance, and *s* is slope of the curve in *x*_50_.

3 $$W = \frac{{W_{{{\text{max}}}} }}{{1 + {\rm{e}}^{{ - k(t - t_{m} )}} }},$$

where, *w* is the absorbance value at time *t*, *k* is a constant that determines the curvature of the growth pattern, and *t*_*m*_ is the inflection point when the absorbance rate reaches the peak. The absorbance value at *t*_m_ is half of its maximum value (*w*_max_).

### Modeling the Effect of Temperature on Solubilizing Ability of Selected Bacteria

Four constant temperatures (4 °C, 18 °C, 30 °C, and 40 °C) were subjected to propose mathematical models for prediction of the beneficial bacterial growth in NB medium during 34 h of incubation. The turbidity of the culture was estimated at 600 nm. Equation (3) is used to quantify the response of potentially beneficial bacterial isolates to temperatures [6]. A modified beta model (Eq. 4) was used to predict the rate of absorbance increase over time (absorbance per hour) at different temperature levels of incubation [24]:

4 $$\begin{array}{*{20}l} {f\left( T \right) = \left( {\frac{{T_{{\text{c}}} - T}}{{T_{{\text{c}}} - T_{{\text{o}}} }}} \right) \left( {\frac{{T - T_{{\text{b}}} }}{{T_{{\text{o}}} - T_{{\text{b}}} }}} \right)^{{\left( {\frac{{T_{{\text{o}}} - T_{{\text{b}}} }}{{T_{{\text{c}}} - T_{{\text{b}}} }}} \right)}} } \hfill & {{\text{if}}\;T > T_{{\text{b}}} \;{\text{and}}\;T < T_{{\text{c}}} } \hfill \\ {f\left( T \right) = 1 } \hfill & { {\text{if}}\;T = T_{{\text{o}}} } \hfill \\ {f\left( T \right) = 0 } \hfill & {{\text{if}}\;T \le T_{{\text{b}}} \;{\text{or}}\;T \le T_{{\text{c}}} } \hfill \\ \end{array} ,$$

where *T* is the temperature, *T*_b_ the base temperature, *T*_o_ the optimum temperature, and *T*_c_ the ceiling temperature. The parameters were estimated by the least-squares method using the non-linear regression procedure and repeated optimization method.

### Data Analysis

Differences among treatment mean (the least significant difference (LSD) test) was calculated using the SPSS software (ver. 16.0 for windows) at a significance level of 0.05. Modeling analyses along with the determination of standard deviation, coefficient of variation, the coefficient of determination, and root-mean-square error as well as the drawing the regression graphs were performed using SigmaPlot software (ver. 14).

## Results

### Isolation of P, K, and Zn Solubilizing and N_2_ Fixing Bacteria

All bacterial isolates were screened after 3 and 7 days of incubation to determine their P, K, and Zn solubilization and N fixation efficiency on the isolation plates. Accordingly, 17, 14, and 4 isolates were able to produce a clear zone around their colonies on the Pikovskaya, Aleksandrov, and Tris-mineral medium, respectively (Table 1). In detail, 18.6%, 15.4%, and 4.4% of isolates were able to solubilize P, K, and Zn from insoluble compounds in solid mediums. There was not any blue zone around isolates colonies on solid N-free medium after 3 days of incubation, but the appearance of blue zone was considered as a qualitative evidence of N_2_ fixation for four isolates after 7 days of incubation (Table 1).

**Table 1:** P, K, and Zn solubilization and N fixation efficiency after 3 and 7 days of incubation

Isolates	PE (%) ± SD		Isolates	KE (%) ± SD	
	3 days	7 days		3 days	7 days
NT9	196 ± 7	200 ± 10	NT6	191 ± 7	192 ± 6
NT10	162 ± 4	186 ± 11	CT21	175 ± 9	192 ± 18
NT6	172 ± 10	183 ± 6	NT15	155 ± 22	188 ± 21
NT28	165 ± 8	181 ± 8	NT28	136 ± 12	185 ± 11
CT63	160 ± 3	174 ± 9	CT45	142 ± 7	181 ± 12
CT30	141 ± 8	172 ± 15	CT64	175 ± 1	178 ± 19
CT34	142 ± 15	166 ± 7	CT16	146 ± 14	173 ± 6
CT35	150 ± 10	164 ± 5	CT67	159 ± 3	171 ± 7
CT32	161 ± 2	161 ± 5	CT44	144 ± 6	166 ± 17
CT15	147 ± 6	160 ± 18	NT1	166 ± 8	165 ± 5
CT59	135 ± 4	150 ± 6	CT9	147 ± 14	158 ± 4
NT1	136 ± 5	142 ± 8	NT10	155 ± 9	155 ± 1
CT5	134 ± 2	136 ± 7	CT34	152 ± 3	154 ± 3
NT5	–	134 ± 4	NT9	152 ± 8	143 ± 4
CT13	123 ± 6	131 ± 7			
NT29	–	121 ± 2			
NT20	–	118 ± 6			

Isolates	ZnE (%) ± SD		Isolates	NE (%) ± SD	
NT6	233 ± 12	250 ± 23	NT3	–	136 ± 5
NT1	214 ± 16	229 ± 21	NT2	–	123 ± 3
NT10	212 ± 12	224 ± 18	NT31	–	117 ± 1
CT34	205 ± 14	213 ± 13	CT43	–	115 ± 1

SD: standard deviation; PE, KE, and ZnE: P, K, and Zn solubilization efficiency, respectively; NE: nitrogen fixation efficiency, NT: no tillage, CT: conventional tillage

**Fig. 1:**
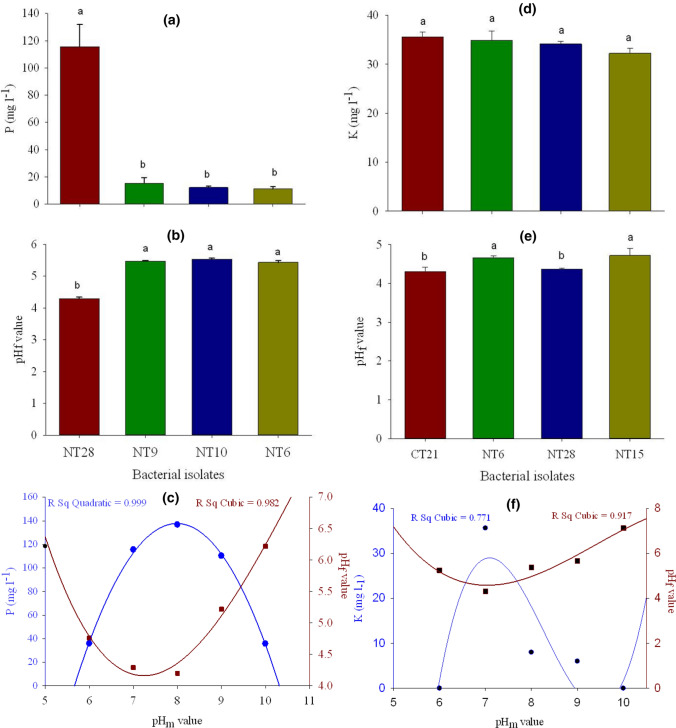
Quantitative estimation of P solubilization (mg l^−1^) (**a**); pH of PVK medium after the 14 days of incubation (pH_f_) at starting pH of 7 (pH_m_) (**b**); relationship between the pH_m_ and pH_f_ and solubilized P (mg l^−1^) by PSB strain (NT28) (**c**). Quantitative estimation of K solubilization (mg l^−1^) (**d**); pH_f_ of Aleksandrov medium at pH_m_ of 7 (**e**); relationship between the pH_m_ and pH_f_ and solubilized K (mg l^−1^) by KSB strain (CT21) (**f**). Means followed by the same letter(s) are not significantly different based on the least significant difference (LSD) test at 0.05 probability level

**Fig. 2:**
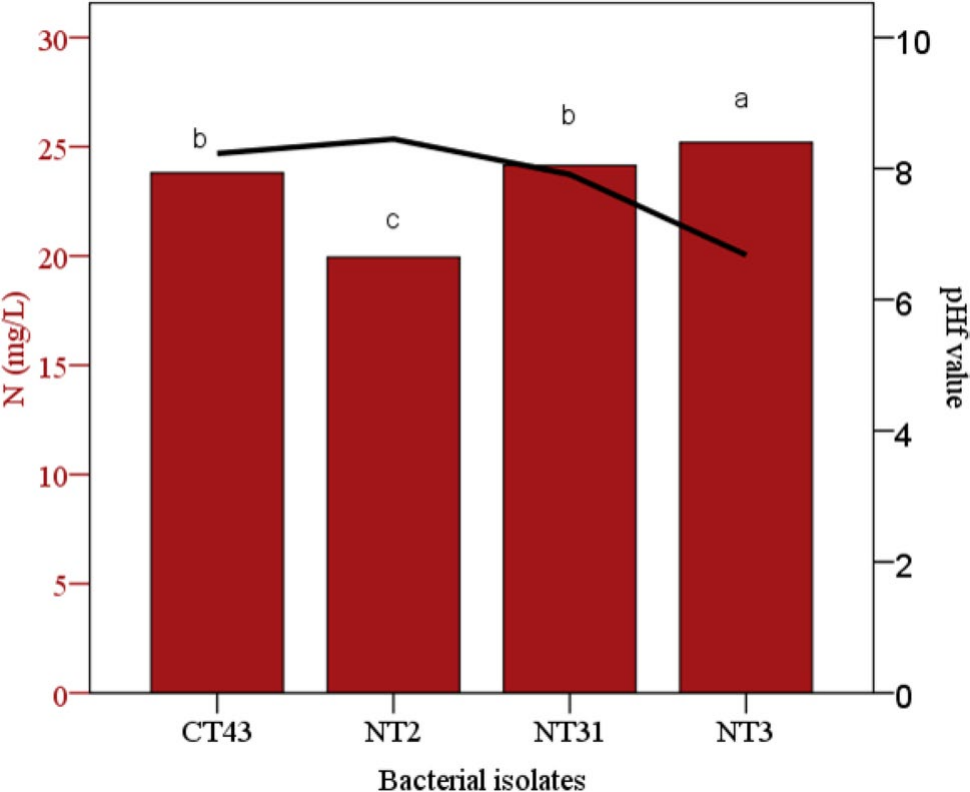
Quantitative estimation of N_2_ fixation (mg l^−1^) by NFB strain (NT3) activity and final pH of N-free semi-solid medium after the 14 days of incubation (pH_f_). Means followed by the same letter are not significantly different based on the least significant difference (LSD) test at 0.05 probability level

The results of further examination of the potentially beneficial bacterial solubilization and fixation activity in the corresponding liquid medium, after 14 days of inoculation, are presented in Figs. 1, 2, and Table 2. In this regard, the amounts of P release from insoluble phosphate compound by the four best phosphate-solubilizing bacteria (PSB) ranged from 11.1 to 115.5 mg l^−1^. Isolate NT28 had significantly higher P-solubilization ability as compared to other isolates (NT9, NT10, and NT6) (Fig. 1a)*.*

**Table 2:** Values of Zn solubilization (mg l^−1^) determined at different pH levels of liquid Tris-mineral medium

Isolates	pH_m_ = 6		pH_m_ = 7		pH_m_ = 8		pH_m_ = 9		pH_m_ = 10	
	Zn (SD)	pH_f_ (SD)	Zn (SD)	pH_f_ (SD)	Zn (SD)	pH_f_ (SD)	Zn (SD)	pH_f_ (SD)	Zn (SD)	pH_f_ (SD)
NT6	0	6.52^a^ (0.12)	0	6.78^a^ (0.07)	1.21^c^ (0.23)	7.12^ab^ (0.11)	91.80^b^ (21.73)	7.32^a^ (0.12)	0	9.12^ab^ (0.19)
NT1	0	6.82^a^ (0.19)	0	6.76^a^ (0.08)	37.01^a^ (6.54)	6.95^b^ (0.09)	3.20^c^ (0.54)	7.93^a^ (0.04)	0	8.86^b^ (0.24)
NT10	0	6.62^a^ (0.12)	0	6.56^b^ (0.20)	17.09^b^ (4.63)	7.15^ab^ (0.16)	389.90^a^ (42.43)	6.98^a^ (0.13)	126.82 (54.51)	7.38^c^ (0.17)
CT34	0	6.55^a^ (0.04)	0	6.69^ab^ (0.17)	0	7.33^a^ (0.07)	1.11^c^ (0.31)	7.76^a^ (0.15)	0	9.36^a^ (0.11)

pH_m_: the pH of medium culture before the incubation period; pH_f_: the pH of medium culture after the 14 days of incubation period; Zn: zinc solubilization activity (mg l^−1^); SD: standard deviation

Means in each column followed by the same letter(s) are not significantly different based on the least significant difference (LSD) test at 0.05 probability level

The amount of K released by potassium-solubilizing bacteria (KSB) ranged from 32.2 (isolate NT15) to 35.6 (isolate CT21) mg l^−1^, which were not significantly different from each other (Fig. 1d). The maximum solubilization occurred when the KSB activities significantly decreased the pH of the medium during the incubation (Fig. 1e). Four NFB isolates showed biological N_2_ fixation activity in liquid N-free medium in a range of NH_4_-N from 19.9 to 25.2 µg ml^−1^. The highest N_2_ fixation ability was belonged to NT3 isolate which was significantly higher than the other isolates (Fig. 2).

The results showed that the Zn-solubilizing bacteria (ZSB) activities were affected by the pH_m_ of the Tris-mineral medium. All four ZSB isolates did not have solubilization activity at pH 6 and 7, while Zn solubilization was observed in the range of pH 8–10 (Table 2), although these isolates were able to produce a clear zone around their colonies on Tris-mineral agar medium at pH 7 (data not shown). The highest Zn solubilization (389.9 mg l^−1^) was observed at pH 9 for isolate NT10, which was significantly higher than other isolates. A decrease in the pH of the Tris-mineral medium after 14 days of incubation was observed at starting pH values from 7 to 10, and the final pH of the medium increased to 6.5–6.8 when the incubation pH was 6.0 (Table 2).

**Fig. 3:**
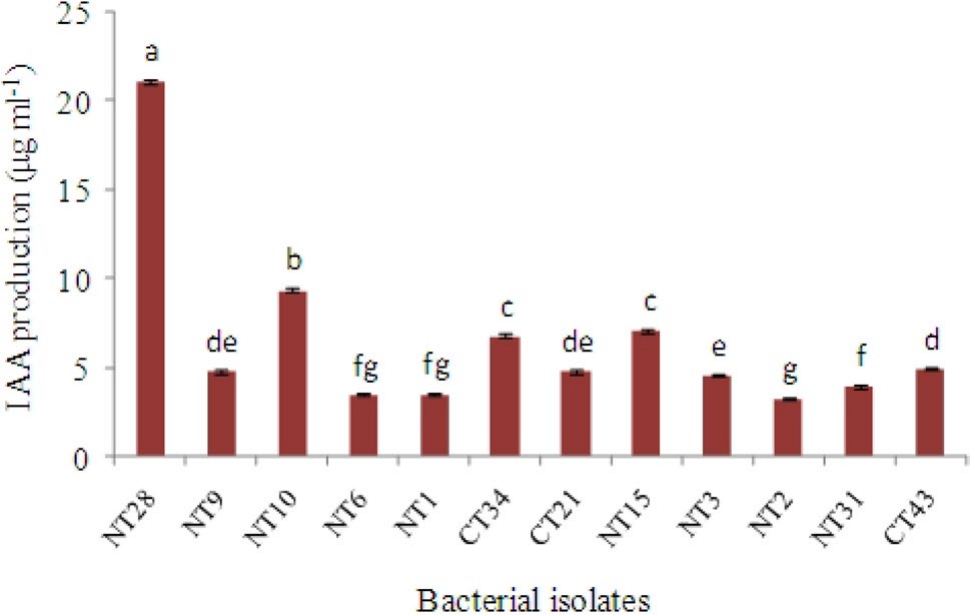
IAI production with and without l-tryptophan (μg ml^−1^) after 72 h of incubation. Means followed by the same letter(s) are not significantly different based on the least significant difference (LSD) test at 0.05 probability level

There were also significant differences among the capacity of these isolates to produce IAA in the presence of l-tryptophan. Almost all strains produced amounts of IAA from 3 to 8 µg ml^−1^, while only one isolate (NT28) showed a very high capacity to 21 µg ml^−1^ (Fig. 3).

One of the strains for each experiment with the highest solubilization and fixation efficiency (NT28, CT21, NT10, and NT3, as PSB, KSB, ZSB, and NFB, respectively) was used for further analysis such as regression analysis and mathematical models. According to the results, cubic and quadratic regressions significantly fitted the relationship between the pH of PVK medium before the incubation (pH_m_) and two other parameters that are the pH of medium after incubation period (pH_f_) and solubilized P by NT28 isolate activity, respectively (Fig. 1c). As the pH_f_ decreased by NT28 activity, the P solubilizing increased significantly. The maximum P solubilization of NT28 was obtained at pH_m_ 8 (136.6 mg l^−1^), which was 18% and 23% more than that at pH_m_ 7 and 9, respectively. Conversely, its pH_f_ value was 2.1% and 19.6% lower than that in pH_m_ 7 and 9, respectively. In fact, the more the decreasing of pH_f_ in the medium by bacterial activities, the more the solubilizing activity.

Regression of cubic equation models significantly (*P* < 0.01) fit correlations between pH_m_ and pH_f_, as well as pH_m_ and solubilized K; the coefficients of determination (*r*^2^) of the equations were 0.77 and 0.92, respectively. The highest amount of solubilization K belonged to pH_m_ 7, which was significantly higher than that in other pH_m_ (Fig. 1f).

### Molecular Identification of Potentially Beneficial Bacteria and Their Relative Abundance in the Soil

**Fig. 4:**
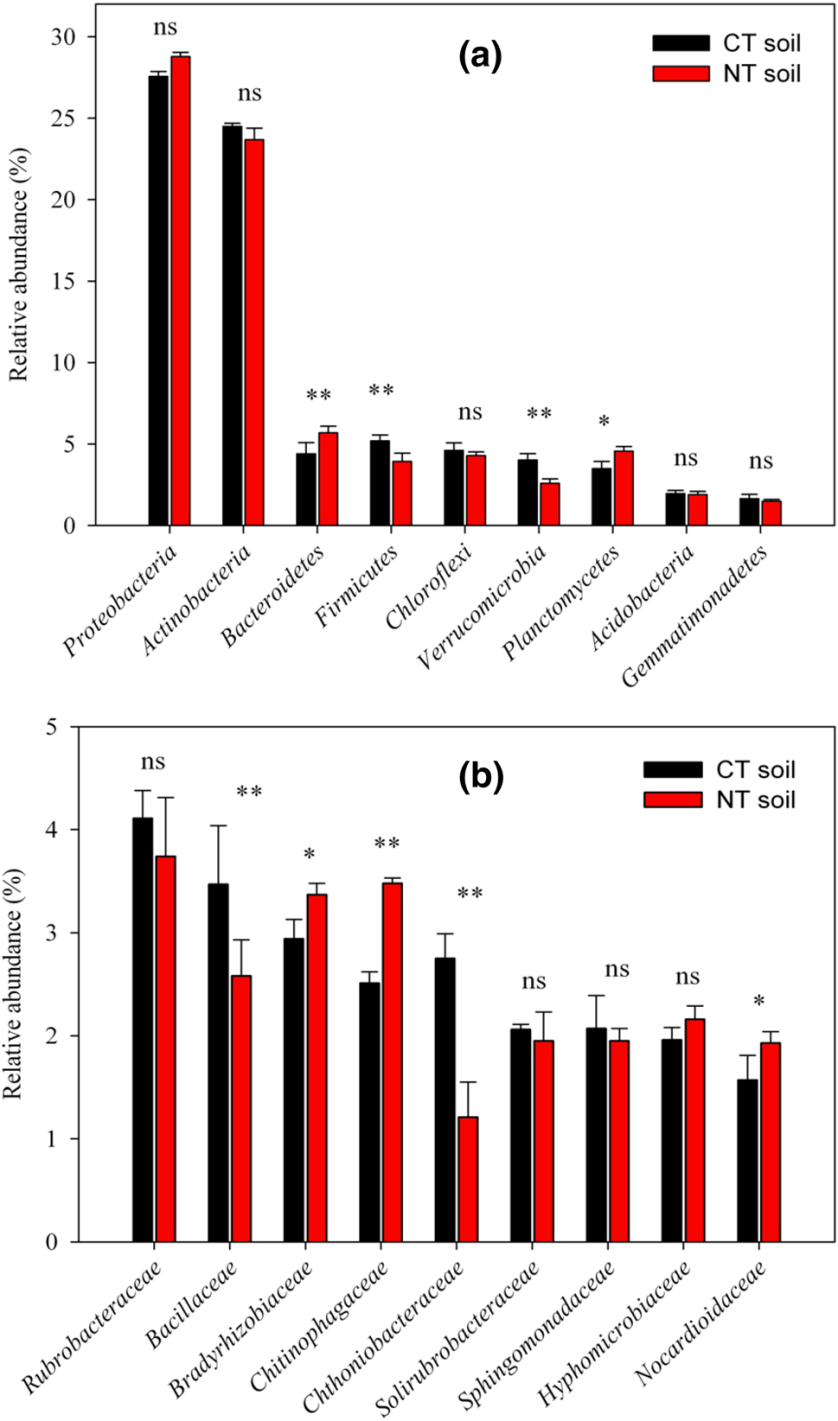
Relative abundance of **a** bacterial phyla (relative abundance > 1%) and **b** families (relative abundance > 2%) for bacterial communities under conventional tillage (CT) and no tillage (NT)

The results of 16S rRNA gene sequencing were compared to those of known 16S rRNA sequences using BLAST and the GenBank database (Table 3). Accordingly, 12 strains belonged to 3 different genera (sequence identity > 98%) including *Pseudomonas*, *Acinetobacter,* and *Comamonas*. the best PSB, KSB, ZSB, and NFB strains were *Acinetobacter pittii*, *Acinetobacter oleivorans*, *Acinetobacter calcoaceticus*, and *Comamonas testosteroni*, respectively (Table 3). Relative abundances (%) of bacterial Phyla (> 1%) are given in Fig. 4a. The most abundant phylum in the CT and NT soil samples was *Proteobacteria*, equal to 27.5 and 27.8% of the total sequences, followed by *Actinobacteria* (24.5 and 23.7%), respectively. Among the less represented phyla, *Firmicutes* and *Verrucomicrobia* were more abundant in CT plots as compared to the NT soils, while *Bacteroidetes* and *Planctomycetes* were significantly more present in the NT soil samples (Fig. 4a). Nine abundant families with a relative abundance >2% were detected in soil samples (Fig. 4b). Accordingly, *Bacillaceae* and *Chthoniobacteraceae* were significantly more abundant in the CT soils as compared to the NT soils. Conversely, the significantly greater relative abundance of *Bradyrhizobiaceae, Chitinophagaceae*, and *Nocardioidaceae* detected in the NT samples (Fig. 4b).

**Table 3:** Identification of selected PGPB isolates based on 16S rRNA gene sequencing, and the genome sequences of the authentic type strains of the corresponding species

Bacterial isolates	Isolation source	Activity in culture medium as	NCBI accession no.	Identified as		
				Type strain	Published species designation (suggested designation)	Similarity (%)
NT28	NT soil	PSB and KSB	MT974044	DSM 21653^T^	*Acinetobacter pittii*	99.7
NT9	NT soil	PSB	MT974040	JCM 5962^T^	*Pseudomonas aeruginosa*	98.9
NT10	NT soil	PSB and ZSB	MT974039	NCCB 22016^T^	*Acinetobacter calcoaceticus*	99.1
NT6	NT soil	PSB, KSB, and ZSB	MT974053	ATCC 14235^T^	*Pseudomonas resinovorans*	99.1
NT1	NT soil	KSB, ZSB	MT974045	ATCC 14235^T^	*Pseudomonas resinovorans*	99.1
CT34	CT soil	ZSB	MT974036	ATCC 14235^T^	*Pseudomonas resinovorans*	99.1
CT21	CT soil	KSB	MT974043	DR1^T^	*Acinetobacter oleivorans*	98.2
NT15	NT soil	KSB	MT974038	DR1^T^	*Acinetobacter oleivorans*	99.6
NT3	NT soil	NFB	MT974042	KS 0043^T^	*Comamonas testosteroni*	99.9
NT2	NT soil	NFB	MT974041	ATCC 14235^T^	*Pseudomonas resinovorans*	99.0
NT31	NT soil	NFB	MT974049	ATCC 14235^T^	*Pseudomonas resinovorans*	99.0
CT43	CT soil	NFB	MT974037	DSM 50071^T^	*Pseudomonas aeruginosa*	95.6

The comparison of the presence of phylum, families, and genera where our potentially beneficial bacteria belong to, in NT and CT soil samples, are presented in Table 4. All 12 bacterial strains belonged to the *Proteobacteria* phylum and three families (*Moraxellaceae*, *Pseudomonadaceae*, and *Comamonadaceae*), which were more present in the NT samples as compared to the CT, although only significantly for *Pseudomonadaceae* (*P* < 0.01)*.* Similarly, at the genera level, the presence of *Pseudomonas* and *Comamonas* genera in NT was significantly higher when compared to the CT (Table 4).

**Table 4:** Comparison of the presence of beneficial bacteria at phylum, family, and genus levels they belong to, in soil under conventional tillage (CT) and no tillage (NT) using paired *t* test

Phylum	NT	CT	*T* value	Family	NT	CT	*T* value	Genera	NT	CT	*T* value
				*Moraxellaceae*	0.017 ± 0.016	0.014 ± 0.008	0.68 ns	*Acinetobacter*	0.003 ± 0.000	0.001 ± 0.000	1.64^ns^
*Proteobacteria*	28.78 ± 0.31	27.56 ± 0.26	1.20 ns	*Pseudomonadaceae*	0.51 ± 0.077	0.08 ± 0.052	20.33**	*Pseudomonas*	0.087 ± 0.008	0.036 ± 0.007	7.10**
				*Comamonadaceae*	1.585 ± 0.076	1.420 ± 0.034	2.85 ns	*Comamonas*	0.160 ± 0.032	0.047 ± 0.020	4.64*

*ns* not significant

* and **Significant at *P* < 0.05 and *P* < 0.01 levels, respectively

The number of 410,128 high-quality sequences was detected with a 300-bp read length. Good’s coverage rate (0.86) showed that the number of sequencing reads was sufficient to estimate bacterial diversity (Table 5). There was no significant difference between CT and NT samples in terms of the Simpson index, while the rate of Chao1 estimator and Shannon index in the NT was significantly higher than those in the CT samples (Table 5).

**Table 5:** Comparison of *α*-diversity indexes (means ± standard error) of bacterial communities under conventional tillage (CT) and no tillage (NT) using paired *t* test (sequence count: 10,000)

Samples	CHAO1 ±	Shannon	Simpson	Goods-coverage
CT soil	4544.27 ± 276.05	9.81 ± 0.12	0.99 ± 0.01	0.86 ± 0.00
NT soil	4852.22 ± 64.94	9.94 ± 0.04	0.99 ± 0.00	0.86 ± 0.00
T value	6.69*	6.75*	1.00 ns	0.22 ns

*ns* not significant

*Significant at *P* < 0.05 levels

### Bacterial Growth Under Different NaCl Concentrations

The growth of the bacterial strains was influenced by different salinity levels under in vitro conditions. The NaCl concentration required for 50% inhibition of absorbance (*X*_50_) was about 2.6–3.7%. The maximum bacterial growth (Ab_max_) was observed in a range of 0.6% (KSB strain)–0.92% (NFB strain) NaCl concentration, which was more than that in control (0.5% NaCl). According to the modeling analyses, a complete inhibition was estimated at 9.3% NaCl for *Comamonas testosteroni* (NT3, NFB strain), which was 91%, 165%, and 51% more than *Acinetobacter oleivorans* (CT21, KSB strain), *Acinetobacter pittii* (NT28, PSB strain), and *Acinetobacter calcoaceticus* (NT10, ZSB strain), respectively (Table 6A). The maximum rate of absorbance (*W*_max_) under different salt concentrations and after 48 h of incubation is presented in Table 6. Accordingly, the maximum growth belonged to *Comamonas testosteroni*, NFB strain, in each NaCl concentration. The time (hours) required to provide the ending of the linear stage is around 24–27 h for KSB strain, 18–22 h for PSB strain, 21–23 h for ZSB strain, and 20–22 h for NFB strain, at different NaCl concentrations, where this inflection point indicated the half of its maximum value (*w*_max_) (Table 6B).

**Table 6:** Effects of different NaCl concentrations on the PGPB growth

Parameters		KSB isolate (CT21)	PSB isolate (NT28)	ZSB isolate (NT10)	NFB isolate (NT3)
(A)					
Ab_max_ ± SE		0.67 ± 0.03	0.78 ± 0.06	0.64 ± 0.94	0.92 ± 0.01
* x*_50_ ± SE		2.58 ± 0.13	2.65 ± 0.26	2.77 ± 0.17	3.68 ± 0.01
* s* ± SE		4.83 ± 1.11	3.50 ± 1.36	6.04 ± 2.39	9.26 ± 1.75
*R*^2^		0.98	0.94	0.95	0.99

NaCl concentrations	Parameters				
(B)					
0.5%	*W*_max_ ± SE	0.74 ± 0.03	0.79 ± 0.04	0.70 ± 0.02	0.81 ± 0.07
	*k* ± SE	0.13 ± 0.01	0.14 ± 0.02	0.14 ± 0.01	0.12 ± 0.02
	*t*_*m*_ ± SE	24.60 ± 1.09	19.35 ± 1.28	22.39 ± 0.80	21.84 ± 1.96
	*R*^2^	0.99	0.98	0.99	0.97
1%	*W*_max_ ± SE	0.65 ± 0.02	0.73 ± 0.03	0.61 ± 0.02	0.81 ± 0.05
	*k* ± SE	0.14 ± 0.01	0.13 ± 0.01	0.16 ± 0.01	0.14 ± 0.02
	*t*_*m*_ ± SE	24.37 ± 0.69	20.10 ± 1.21	21.66 ± 0.71	20.22 ± 1.59
	*R*^2^	0.99	0.99	0.99	0.98
1.5%	*W*_max_ ± SE	0.60 ± 0.02	0.61 ± 0.02	0.59 ± 0.02	0.78 ± 0.05
	*k* ± SE	0.15 ± 0.01	0.16 ± 0.01	0.16 ± 0.01	0.15 ± 0.02
	*t*_*m*_ ± SE	24.03 ± 0.72	18.73 ± 0.70	21.84 ± 0.74	19.89 ± 1.53
	*R*^2^	0.99	0.99	0.99	0.98
2%	*W*_max_ ± SE	0.57 ± 0.03	0.61 ± 0.02	0.58 ± 0.01	0.82 ± 0.08
	*k* ± SE	0.13 ± 0.01	0.15 ± 0.02	0.16 ± 0.01	0.15 ± 0.02
	*t*_*m*_ ± SE	26.89 ± 1.22	19.08 ± 0.91	21.73 ± 0.54	21.49 ± 1.48
	*R*^2^	0.99	0.99	0.99	0.98
3%	*W*_max_ ± SE	0.22 ± 0.02	0.29 ± 0.02	0.25 ± 0.01	0.33 ± 0.02
	*k* ± SE	0.10 ± 0.02	0.13 ± 0.02	0.14 ± 0.01	0.19 ± 0.03
	*t*_*m*_ ± SE	23.88 ± 3.05	21.46 ± 1.52	21.25 ± 0.86	20.85 ± 1.22
	*R*^2^	0.96	0.98	0.99	0.98

Estimation after 2 days of incubation as calculated by Eq. 2 (A); prediction of absorbance rate per hour during 2 days of incubation at different salinity levels of nutrient broth medium, as calculated by model Eq. 3 (B)

Ab_max_: maximum absorbance; *x*_50_: concentration of NaCl required for 50% inhibition of the maximum absorbance; *s*: slope of the curve in *x*_50_; R^2^: coefficient of determination; *W*: the absorbance value at time *t*; *k*: a constant that determines the curvature of the growth pattern; *t*_*m*_: the inflection point when the absorbance rate reaches the peak

### Bacterial Growth Under Different Temperatures

Since all biological processes are affected by temperature, the growth of these bacterial strains was estimated at different temperatures using Eq. 4 and characterized by three cardinal temperatures including base (*T*_b_), optimum (*T*_o_), and ceiling (*T*_c_) temperatures. The minimum (*T*_b_) and maximum (*T*_c_) temperatures, estimated by the model, were 2.8 and 45.1 °C for *Acinetobacter oleivorans* (KSB strain), 2 and 44.6 °C for *Acinetobacter pittii* (PSB strain), 2.3 and 46 °C for *Acinetobacter calcoaceticus* (ZSB strain), and 2.1 and 42.18 °C for *Comamonas testosteroni* (NFB strain), respectively. Also, the estimated optimal growth temperatures (*T*_o_) were 24.6, 28.2, 27.4, and 23.3 °C, respectively (Table 7A). The maximum absorbance rates (*W*_max_) after 34 h of incubation at different temperatures are given in Table 7. The inflection point of the function, where the function changes concavity, was widely ranged from 17.9 h to 26.2 h at 4 °C, 16.8 h to 19.5 h at 18 °C, 12.1 h to 16.6 h at 30 °C, and 17 h to 23.8 h at 40 °C, respectively (Table 7B).

**Table 7:** Prediction of the absorbance rate (per hour) at different temperatures of incubation, as calculated by Eq. 4 (A) and estimation of the effect of different temperatures on bacterial growth during 34 h of incubation, as calculated by model Eq. 3 (B)

Parameters		KSB isolate (CT21)	PSB isolate (NT28)	ZSB isolate (NT10)	NFB isolate (NT3)
(A)					
* T*_o_ ± SE		24.56 ± 1.71	28.25 ± 0.91	27.41 ± 1.32	23.28 ± 1.88
* T*_b_ ± SE		2.82 ± 0.22	2.02 ± 0.14	2.32 ± 0.19	2.06 ± 0.24
* T*_c_ ± SE		45.12 ± 1.11	44.56 ± 0.00	46.05 ± 1.13	42.18 ± 1.83
*R*^2^		0.95	0.97	0.92	0.95

Temperatures	Parameters				
(B)					
4 °C	*W*_max_ ± SE	0.14 ± 0.04	0.11 ± 0.01	0.10 ± 0.01	0.13 ± 0.06
	*k* ± SE	0.12 ± 0.02	0.14 ± 0.04	0.15 ± 0.03	0.10 ± 0.03
	*t*_*m*_ ± SE	26.25 ± 5.41	17.88 ± 2.91	22.89 ± 2.27	26.49 ± 9.79
	*R*^2^	0.99	0.95	0.98	0.94
18 °C	*W*_max_ ± SE	0.52 ± 0.01	0.72 ± 0.07	0.55 ± 0.02	0.64 ± 0.02
	*k* ± SE	0.29 ± 0.02	0.18 ± 0.04	0.23 ± 0.02	0.28 ± 0.03
	*t*_*m*_ ± SE	19.07 ± 0.33	19.50 ± 1.63	18.39 ± 0.47	16.83 ± 0.50
	*R*^2^	0.99	0.97	0.99	0.99
30 °C	*W*_max_ ± SE	0.49 ± 0.05	0.58 ± 0.01	0.45 ± 0.01	0.51 ± 0.02
	*k* ± SE	0.15 ± 0.04	0.26 ± 0.02	0.25 ± 0.03	0.18 ± 0.02
	*t*_*m*_ ± SE	16.59 ± 2.22	12.14 ± 0.55	12.38 ± 0.51	13.66 ± 0.86
	*R*^2^	0.96	0.99	0.99	0.99
40 °C	*W*_max_ ± SE	0.45 ± 0.08	0.62 ± 0.07	0.40 ± 0.01	0.18 ± 0.01
	*k* ± SE	0.16 ± 0.04	0.18 ± 0.05	0.26 ± 0.03	0.20 ± 0.02
	*t*_*m*_ ± SE	23.83 ± 3.05	20.62 ± 2.04	17.01 ± 0.53	18.53 ± 0.84
	*R*^2^	0.97	0.96	0.99	0.99

*T*: the temperature; *T*_b_: the base temperature; *T*_o_: the optimum temperature; *T*_c_: the ceiling temperature; R^2^: coefficient of determination; *W*: the absorbance value at time *t*(34 h); *k*: a constant that determines the curvature of the growth pattern; *t*_*m*_: the inflection point when the absorbance rate reaches the peak which is half of its maximum value (*w*_max_)

## Discussion

Since the composition of potentially beneficial bacterial communities and their colonization capacity in soil are affected by management practices such as tillage [25], the bacterial isolation was performed at a field with two different tillage practices including CT and NT. The present study showed that most of beneficial bacterial isolates (81% of the isolates with the best plant growth-promoting traits such as higher solubilization and fixation efficiency) belonged to the no-tillage soil. This finding suggests that the PGPB colonization could be enhanced under NT practice [25] because NT causes slow changes in microbial composition communities [26]. In this regard, Torabian et al. [14] reported that N_2_ fixation in some crops such as soybean and chickpea were greater under NT than that under CT.

According to the results, several bacterial isolates have been isolated from the durum wheat field with the remarkable capability to solubilize of insoluble inorganic P, K, and Zn compounds and fix N_2_. Twelve isolates expressing some plant growth-promoting traits (i.e., P, K, and Zn solubilization, N_2_ fixation, and IAA production) at the highest levels were further characterized in liquid mediums and identified. Our PSB and KSB isolates identified as *Acinetobacter pittii*, *Pseudomonas alcaligenes*, *Acinetobacter calcoaceticus*, *Acinetobacter* sp., and *Acinetobacter oleivorans.* Although bacteria belonging to *Pseudomonas* have been already reported to include PSB and KSB as well as *Acinetobacter* which is known as PSB [27–29], to our knowledge, this is the first report on K solubilization potential of genera *Acinetobacter*.

A high regression coefficient was observed between the reduction in pH_f_ and increscent capacity to solubilize P in liquid medium after 14 days of incubation. All the pH_m_ of cultures showed a shift in pH_f_ towards the acidic range, and this can give a clue that organic acid exudation might be involved in P solubilizing at all pH_m_. Accordingly, the PSB strain (NT28) was a more efficient phosphate solubilizer at pH_m_ 8 than at other pH_m_, while the lowest pH_f_ was obtained from pH_m_ 7. These findings are inconsistent with other studies, such as Bakhshandeh et al. [6] for *Pantoea ananatis*, *Rahnella aquatilis*, and *Enterobacter* sp., who reported that the highest levels of P solublilizing activity were observed at the optimum pH (7.0). A possible explanation is that this strain was isolated from a soil with a quite high pH (7.5), a common situation for soils from southern Italy whose pH are often about 8 [30].

The K solubilizing activity was observed at pH_m_ of 7–9, equal to 35.6, 8, and 6 µg ml^−1^ along with pH_f_ of 4.3, 5.4 and 5.7, respectively. In fact, more activity of KSB strain resulted in a further decrease in pH_f_ and consequently, more K releasing from mica compound. Similar to the PSB strategy, it may be related to releasing organic acids in the medium by KSB strain, *Acinetobacter oleivorans*. Such results are confirmed by Bakhshandeh et al. [19] and Yaghoubi et al. [11] who stated that the various types of organic acids produced due to the metabolism of KSB isolates which can affect mica dissolution by decreasing the pH of the environment, forming frame work-destabilizing surface complexes or by complexing metals in solution. Likewise, polysaccharides can attach to the mineral surface by adsorbing the organic acids and consequently can increase the organic acids concentration near the mineral [31].

The four ZSB isolates in this research were identified as *Acinetobacter calcoaceticus*, *Pseudomonas alcaligenes,* and *Pseudomonas* sp. It has also been reported that the majority of ZSB belongs to *Pseudomonas*, *Acinetobacter*, *Bacillus*, *Enterobacter*, *Xanthomonas*, and *Stenotrophomonas* genera [18, 32]. The ability of ZSB isolates including *Pseudomonas* sp. and *Bacillus* sp. was assessed by Saravanan et al. [33] who reported a maximum Zn solubilization equal to 16.4 µg g^−1^of Zn in the ZnO. Similarly, Zn-solubilizing potentiality of 143 ZSB isolates was assessed by Gandhi and Muralidharan [18] and reported the maximum ZSB activity by *Acinetobacter* sp. equal to 36.5 µg Zn ml^−^ of medium amended by ZnO.

Since soil pH is a relevant factor affecting the colonization, abundance, and prevalence of PGPB in the soil [25], attempt was made to investigate the effect of different pH values of the medium (pH_m_) on beneficial bacterial activity in vitro. In this regard, the responses of bacterial strains to different pH_m_ values were different. For instance, the highest Zn solubilizing activity was found at pH_m_ 8–10, while no solubilization was observed at pH_m_ of 6 and 7. On the other hand, as above discussed, the maximum solubilization of K and P occurred at pH_m_ 7 and 8, respectively. A similar finding for ZSB has been reported in our previous research; accordingly, the highest ZSB activity was observed at pH_m_ 9, which was significantly higher than that at other pH [13]. It has already reported that some PGPB are able to mobilize mineral nutrients even at pH 12 through a variety of mechanisms such as production of bacterial metabolites [34]. In this regard, Mimmo et al. [35] reported that mobilization of mineral nutrients like Zn by low-molecular weight organic acids, phenolics, and siderophores is mainly caused by the complexing capacity of these molecules rather than their acidity. This complexing capacity is increased at higher pH because deprotonated carboxilic and phenolic moieties are better Lewis bases to react with metal cations (Lewis acids). It has also been stated that organic compounds with more than one acidic moiety (e.g., citric acid) have a stronger complexing capacity at higher pH. when all the acidic functional groups are successively deprotonated thus allowing the formation of polydentate complexes with cations possessing more than one positive charge like Fe^3+^ and Zn^++^ [35].

Therefore, our bacterial strains could be beneficial to use as bio-inoculants on agricultural land with high pH values, since high pH is one of the most important factors contributing to the unavailability of Zn in soils [36]. Furthermore, insoluble Zn compounds such as Zn(OH)_2_ and ZnCO_3_ usually form from soluble Zn compound and Zn chemical fertilizer at pH of 7.7–9.1 [32], which can result in unavailability of Zn in soils and Zn deficiency symptoms in plants, especially on calcareous soils in southern Italy (pH > 8).

In the present research, N_2_ fixation activity in liquid medium was observed from 19.2 µg ml^−1^ (*Pseudomonas* sp., strain NT2 with 123% of N fixation efficiency) to 25.2 µg ml^−1^ (*Comamonas testosteroni,* strain NT3 with 136% of N fixation efficiency). It has been already proved that *Pseudomonas* genusis one of the most important of NFB genera [37], but no studies have been reported that the *Comamonas* genusis capable to fix on N_2_.

The final pH of the medium after the incubation period slightly decreased for NT3 strain, *Comamonas testosteroni*, while inversely the pH_f_ for other NFB strains, *Pseudomonas* sp., increased. NFB bacteria convert the atmospheric N_2_ into ammonia (NH_3_) as a plant-utilizable form [10]. This process is an H^+^ consumer and subsequently leads to change the pH. It has in fact reported that increasing the pH of the rhizospheric soil may be an important consequence of N_2_ fixation by bacteria [38]. On the other hand, Oliveira et al. [39] examined five NFB bacterial strains and reported the pH of the culture medium after growth for four strains was neutral while another strain changed the pH to acidic.

The differences in pH_f_ among isolates may be related to the different N_2_ fixing systems. Similarly, Ahemad and Kibert [40] stated that N_2_ fixing system varies among different bacterial genera. It has been reported that the N_2_ fixation process is carried out by the nitrogenase complex enzyme, including a dinitrogenase reductase and dinitrogenase [41]. Dinitrogenase reductase provides electrons with high reducing power while dinitrogenase uses these electrons to reduce N_2_ to NH_3_ [40]. According to the metal cofactor, three different N fixing systems have been recognized including Mo-nitrogenase, V-nitrogenase, and Fe-nitrogenase [41]. Most biological N_2_ fixation is carried out by the activity of the Mo-nitrogenase [40].

Since the bacteria survival during growth depends on their ability to adapt to the varying environmental conditions such as temperature and salt stress [6]; in this study, an attempt was made to optimize the growth conditions of selected beneficial bacteria at different NaCl concentrations (%) and temperatures. However, the finding of the present research showed that the selected bacteria were able to grow on NB medium amended with 0.5–3% NaCl at a range of examined temperatures from 4 to 40 °C, but modeling analyses estimated optimal growth conditions for possible practical applications. In this regard, the best growth conditions for *Acinetobacter oleivorans* (KSB strain), *Acinetobacter pittii* (PSB strain), and *Acinetobacter calcoaceticus* (ZSB strain) were obtained at 0.67, 0.74, and 0.64% of NaCl concentration, at 24.6, 28.2, and 27.4 °C and in pH 7, 8, and 9, respectively. Conditions for *Comamonas testosteroni* (NFB strain) were a bit more different from other strains. Accordingly, the best growth for NFB strain was estimated at higher NaCl concentration (0.92%) and lower temperature (23.3 °C,) than other bacterial strains. Complete inhibition of growth was estimated from 3.5% NaCl for PSB strain to 9.3% NaCl for NFB strain. Application of these bacterial strains with great potential of growth and activity in such wide ranges of pH, salinity levels, and temperatures could be useful in soil inoculation, since it is important to maintain high microbial activity in soils [6, 42], where microorganisms are continually challenged by environmental fluctuations [43]. It has already been reported that microbial growth and activity decreased under saline conditions, due to two primary mechanisms including osmotic effect and specific ion effects [42]. The previous study has shown that the complete inhibitions of *Agrobacterium tumefaciens* and *Rhizobium* sp. growth in NB medium were estimated at 4.3 and 6.6% NaCl, respectively [13]. The optimum NaCl concentration for the majority of bacterial isolates was reported at 0.5% NaCl [6]. Bakhshandeh et al. [6] reported that three PSB including *Pantoea ananatis*, *Rahnella aquatilis,* and *Enterobacter* sp. were tolerant to a range of temperature from 5 to 42.7 °C, 12.7–40 °C, and 10.6–43.3 (with optimum 30, 31.4, and 30 °C), respectively. It has also reported that the optimal temperature for N_2_ fixation by *B. japonicum* ranges from 25 to 30 °C and temperatures over or below this range are inhibitory [14].

The analysis of bacterial community composition and *α* diversity are fully discussed in Yaghoubi et al. [44]. Briefly, soils under NT management had more copiotrophic bacteria including *Proteobacteria, Bacteroidetes*, and *Planctomycetes,* which are known for their high sensitivity to physical soil disturbance [45, 46]. Species richness and bacterial diversity, as estimated by Chao1 and Shannon index, respectively, were higher in soils under NT treatment as compared to the CT practice. This finding is in agreement to others, such as Schmidt et al*. *[47] and Hao et al. [48] who reported a decreasing trend of the microbial community richness and diversity in soil under CT practice, due to a reduced substrate richness.

We analyzed the presence of the taxonomic groups our potentially beneficial bacterial strains belonged to, in order to know if tillage/no-tillage management affects their presence in soil, even if it should be considered that, these taxa can also include bacteria without such beneficial abilities. Based on the analysis of the taxonomic composition, our bacterial strains belonged to the *Proteobacteria* phylum and *Moraxellaceae*, *Pseudomonadaceae*, and *Comamonadaceae* families which are known as copiotrophic taxa, based on their life strategies in response to resource availability [49] and soil management, especially physical soil disturbance by tillage [46]. It seems that the reduction of soil disturbance through NT technique can lead to an increase in the content of soil carbon availability [47] and consequently, results in the higher presence of these phylum and families [26] as compared to the CT. Therefore, we can reasonably assume that the preservation of an agro-ecosystem under no disturbance can boost the presence of these beneficial bacterial strains in soil in comparison with the CT management. Similarly, Dong et al. [28] evidenced that the abundance and diversity of KSB decreased after agricultural activities. Souza et al. [50] also reported that the relative abundance of a PGPB order (*Rhizobiales*) increased under NT practice. A recent study emphasized that the importance of the effect of tillage regime on soil physical and chemical conditions that in turn influence the abundance of beneficial microorganisms [51].

## Conclusion

The present research reveals that some effective bacterial strains including *Pseudomonas*, *Acinetobacter,* and *Comamonas* genera, isolated and identified from the durum wheat field, have such a great potential to solubilize the insoluble forms of phosphate, potassium, and zinc as well as to fix N_2_ gas. To our knowledge, this is the first report of the ability of *Comamonas testosteroni* and *Acinetobacter pittii* to fix nitrogen and to solubilize insoluble potassium compound, respectively. A detailed assessment of the tolerance of beneficial bacterial strains to salt stress threshold and different temperatures are presented in this research using mathematical models. So, these strains could be definitively recommended as inoculants to promote plant growth in an agricultural environment in the wide ranges of pH, salinity levels, and temperatures, but more in-depth studies will be needed to confirm these solubilizing and fixing activities.
